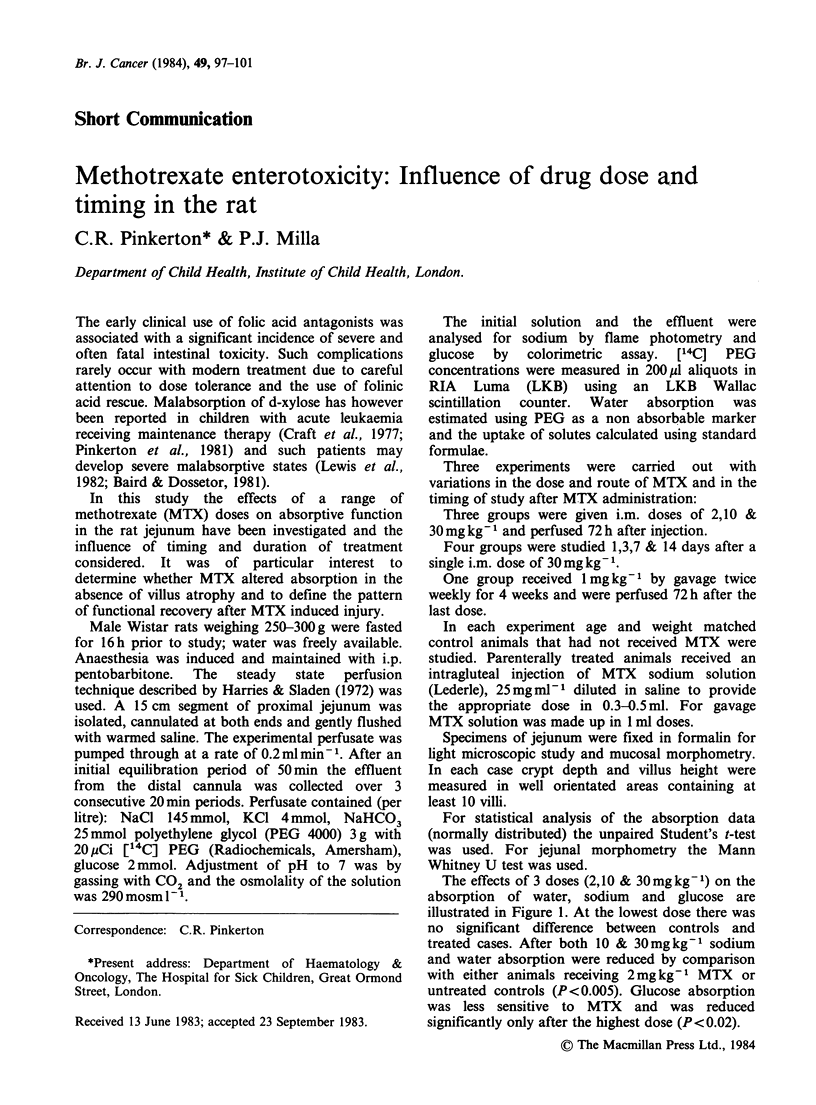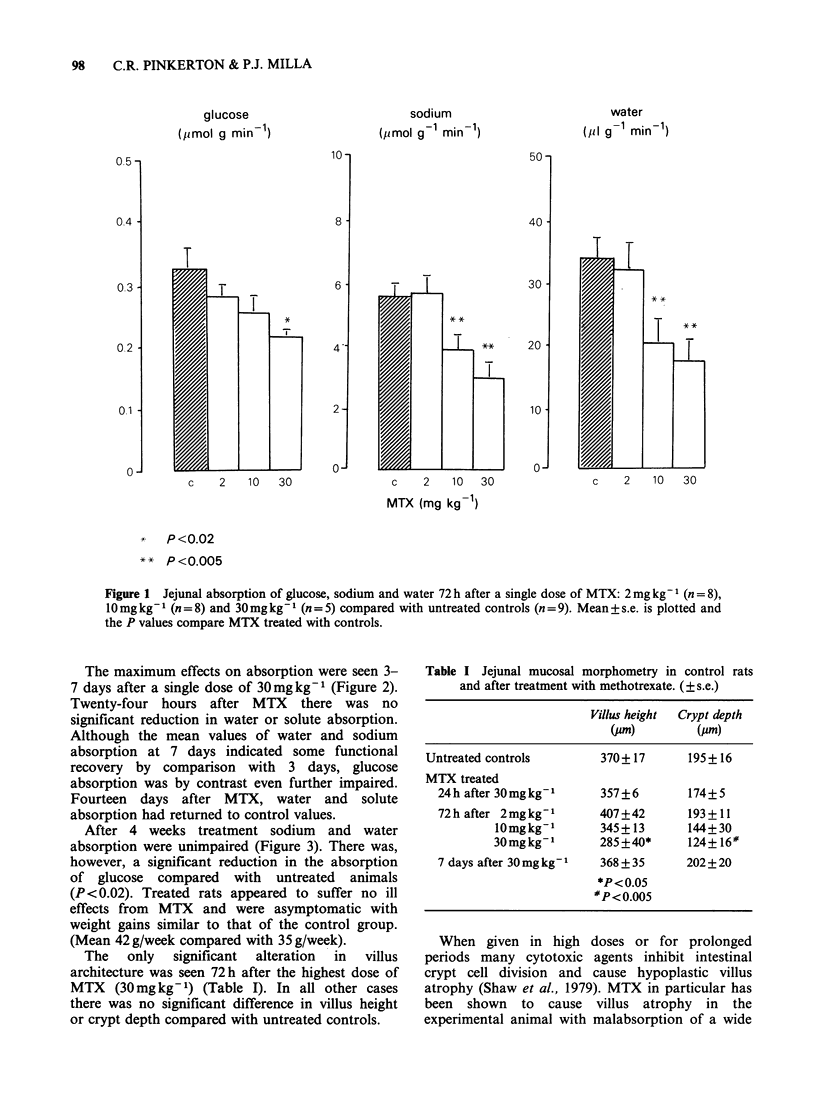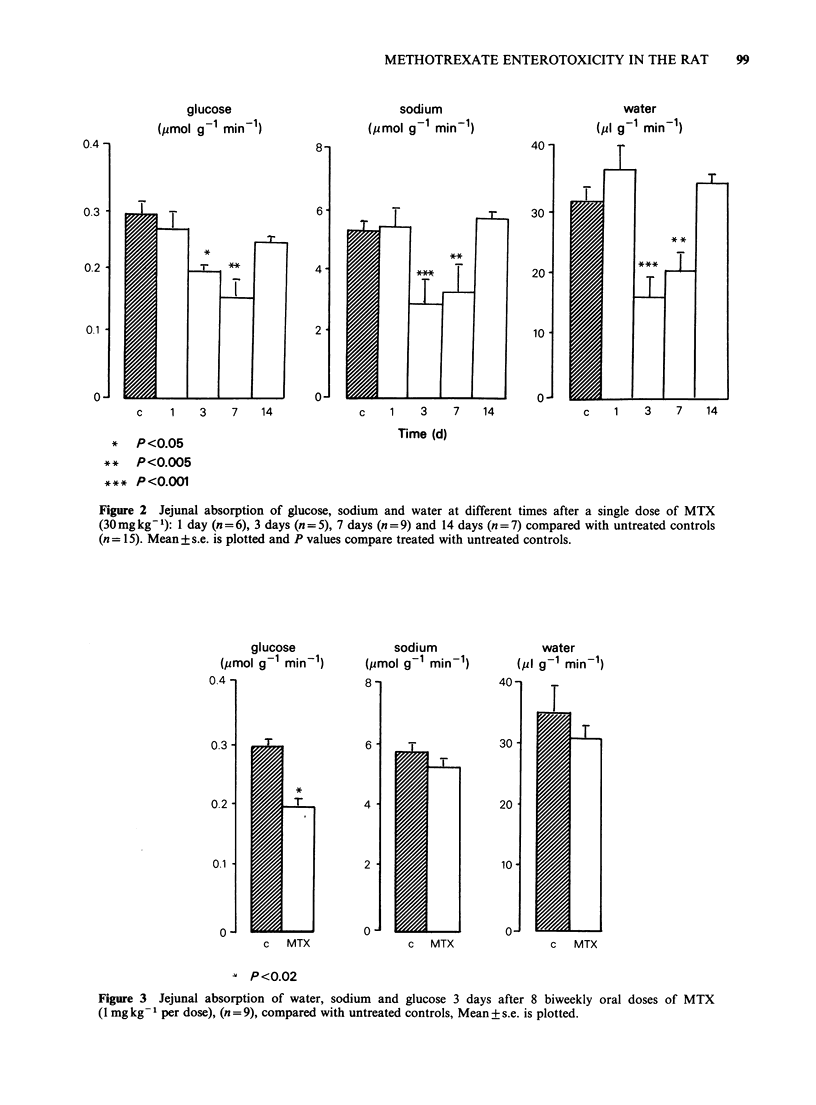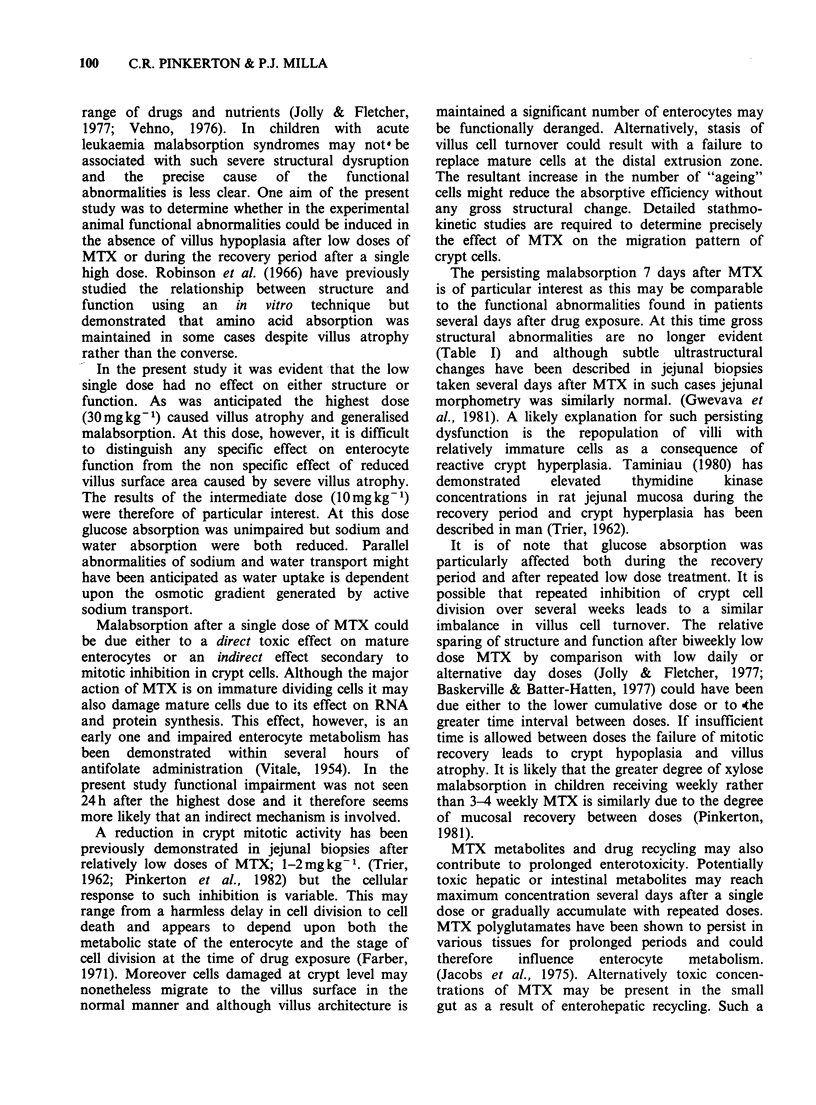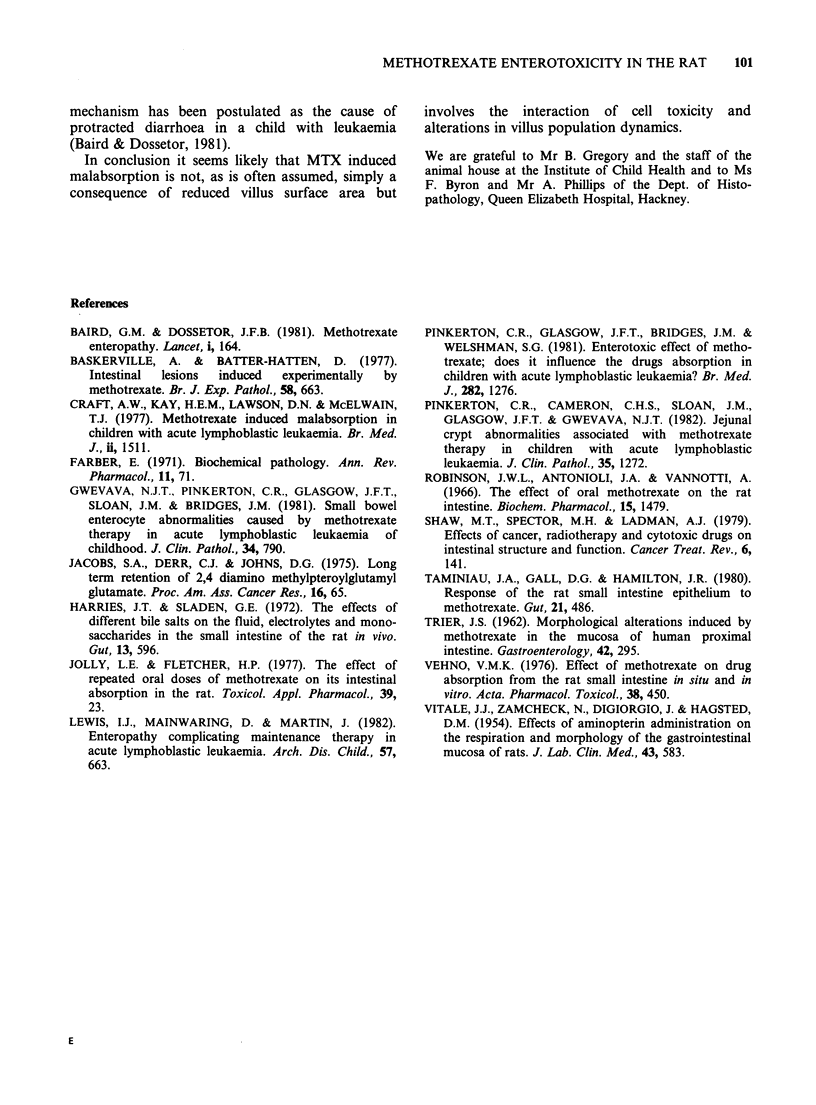# Methotrexate enterotoxicity: influence of drug dose and timing in the rat.

**DOI:** 10.1038/bjc.1984.15

**Published:** 1984-01

**Authors:** C. R. Pinkerton, P. J. Milla


*Br. J. Cancer* (1984), **49**, 97–101

### Short Communication

### Methotrexate enterotoxicity: Influence of drug dose and timing in the rat

C.R. Pinkerton* & P.J. Milla

*Department of Child Health, Institute of Child Health, London.*

The early clinical use of folic acid antagonists was associated with a significant incidence of severe and often fatal intestinal toxicity. Such complications rarely occur with modern treatment due to careful attention to dose tolerance and the use of folinic acid rescue. Malabsorption of d-xylose has however been reported in children with acute leukaemia receiving maintenance therapy (Craft *et al.,* 1977; Pinkerton *et al.,* 1981) and such patients may develop severe malabsorptive states (Lewis *et al.,* 1982; Baird & Dossetor, 1981).

In this study the effects of a range of methotrexate (MTX) doses on absorptive function in the rat jejunum have been investigated and the influence of timing and duration of treatment considered. It was of particular interest to determine whether MTX altered absorption in the absence of villus atrophy and to define the pattern of functional recovery after MTX induced injury.

Male Wistar rats weighing 250–300 g were fasted for 16 h prior to study; water was freely available. Anaesthesia was induced and maintained with i.p. pentobarbitone. The steady state perfusion technique described by Harries & Sladen (1972) was used. A 15 cm segment of proximal jejunum was isolated, cannulated at both ends and gently flushed with warmed saline. The experimental perfusate was pumped through at a rate of 0.2 ml min$^{-1}$. After an initial equilibration period of 50 min the effluent from the distal cannula was collected over 3 consecutive 20 min periods. Perfusate contained (per litre): NaCl 145 mmol, KCl 4 mmol, $NaHCO_3$ 25 mmol polyethylene glycol (PEG 4000) 3 g with 20 $\mu$Ci [$^{14}$C] PEG (Radiochemicals, Amersham), glucose 2 mmol. Adjustment of pH to 7 was by gassing with $CO_2$ and the osmolality of the solution was 290 mosm l$^{-1}$.

Correspondence: C.R. Pinkerton

*Present address: Department of Haematology & Oncology, The Hospital for Sick Children, Great Ormond Street, London.

Received 13 June 1983; accepted 23 September 1983.

The initial solution and the effluent were analysed for sodium by flame photometry and glucose by colorimetric assay. [$^{14}$C] PEG concentrations were measured in 200 $\mu$l aliquots in RIA Luma (LKB) using an LKB Wallac scintillation counter. Water absorption was estimated using PEG as a non absorbable marker and the uptake of solutes calculated using standard formulae.

Three experiments were carried out with variations in the dose and route of MTX and in the timing of study after MTX administration:

Three groups were given i.m. doses of 2,10 & 30 mg kg$^{-1}$ and perfused 72 h after injection.

Four groups were studied 1,3,7 & 14 days after a single i.m. dose of 30 mg kg$^{-1}$.

One group received 1 mg kg$^{-1}$ by gavage twice weekly for 4 weeks and were perfused 72 h after the last dose.

In each experiment age and weight matched control animals that had not received MTX were studied. Parenterally treated animals received an intragluteal injection of MTX sodium solution (Lederle), 25 mg ml$^{-1}$ diluted in saline to provide the appropriate dose in 0.3–0.5 ml. For gavage MTX solution was made up in 1 ml doses.

Specimens of jejunum were fixed in formalin for light microscopic study and mucosal morphometry. In each case crypt depth and villus height were measured in well orientated areas containing at least 10 villi.

For statistical analysis of the absorption data (normally distributed) the unpaired Student's *t*-test was used. For jejunal morphometry the Mann Whitney U test was used.

The effects of 3 doses (2,10 & 30 mg kg$^{-1}$) on the absorption of water, sodium and glucose are illustrated in Figure 1. At the lowest dose there was no significant difference between controls and treated cases. After both 10 & 30 mg kg$^{-1}$ sodium and water absorption were reduced by comparison with either animals receiving 2 mg kg$^{-1}$ MTX or untreated controls ($P < 0.005$). Glucose absorption was less sensitive to MTX and was reduced significantly only after the highest dose ($P < 0.02$).

© The Macmillan Press Ltd., 1984

**Figure 1** Jejunal absorption of glucose, sodium and water 72 h after a single dose of MTX: 2 mg kg$^{-1}$ ($n=8$), 10 mg kg$^{-1}$ ($n=8$) and 30 mg kg$^{-1}$ ($n=5$) compared with untreated controls ($n=9$). Mean ± s.e. is plotted and the $P$ values compare MTX treated with controls.

The maximum effects on absorption were seen 3–7 days after a single dose of 30 mg kg$^{-1}$ (Figure 2). Twenty-four hours after MTX there was no significant reduction in water or solute absorption. Although the mean values of water and sodium absorption at 7 days indicated some functional recovery by comparison with 3 days, glucose absorption was by contrast even further impaired. Fourteen days after MTX, water and solute absorption had returned to control values.

After 4 weeks treatment sodium and water absorption were unimpaired (Figure 3). There was, however, a significant reduction in the absorption of glucose compared with untreated animals ($P<0.02$). Treated rats appeared to suffer no ill effects from MTX and were asymptomatic with weight gains similar to that of the control group. (Mean 42 g/week compared with 35 g/week).

The only significant alteration in villus architecture was seen 72 h after the highest dose of MTX (30 mg kg$^{-1}$) (Table I). In all other cases there was no significant difference in villus height or crypt depth compared with untreated controls.

**Table I** Jejunal mucosal morphometry in control rats and after treatment with methotrexate. (± s.e.)

| | *Villus height* (μm) | *Crypt depth* (μm) |
|---|---|---|
| Untreated controls | 370 ± 17 | 195 ± 16 |
| MTX treated | | |
| 24 h after 30 mg kg$^{-1}$ | 357 ± 6 | 174 ± 5 |
| 72 h after 2 mg kg$^{-1}$ | 407 ± 42 | 193 ± 11 |
| 10 mg kg$^{-1}$ | 345 ± 13 | 144 ± 30 |
| 30 mg kg$^{-1}$ | 285 ± 40* | 124 ± 16# |
| 7 days after 30 mg kg$^{-1}$ | 368 ± 35 | 202 ± 20 |
| | *$P<0.05$ | |
| | #$P<0.005$ | |

When given in high doses or for prolonged periods many cytotoxic agents inhibit intestinal crypt cell division and cause hypoplastic villus atrophy (Shaw *et al.*, 1979). MTX in particular has been shown to cause villus atrophy in the experimental animal with malabsorption of a wide

**Figure 2** Jejunal absorption of glucose, sodium and water at different times after a single dose of MTX (30 mg kg$^{-1}$): 1 day ($n=6$), 3 days ($n=5$), 7 days ($n=9$) and 14 days ($n=7$) compared with untreated controls ($n=15$). Mean ± s.e. is plotted and $P$ values compare treated with untreated controls.

**Figure 3** Jejunal absorption of water, sodium and glucose 3 days after 8 biweekly oral doses of MTX (1 mg kg$^{-1}$ per dose), ($n=9$), compared with untreated controls, Mean ± s.e. is plotted.

range of drugs and nutrients (Jolly & Fletcher, 1977; Vehno, 1976). In children with acute leukaemia malabsorption syndromes may not• be associated with such severe structural dysruption and the precise cause of the functional abnormalities is less clear. One aim of the present study was to determine whether in the experimental animal functional abnormalities could be induced in the absence of villus hypoplasia after low doses of MTX or during the recovery period after a single high dose. Robinson *et al.* (1966) have previously studied the relationship between structure and function using an *in vitro* technique but demonstrated that amino acid absorption was maintained in some cases despite villus atrophy rather than the converse.

In the present study it was evident that the low single dose had no effect on either structure or function. As was anticipated the highest dose ($30\,mg\,kg^{-1}$) caused villus atrophy and generalised malabsorption. At this dose, however, it is difficult to distinguish any specific effect on enterocyte function from the non specific effect of reduced villus surface area caused by severe villus atrophy. The results of the intermediate dose ($10\,mg\,kg^{-1}$) were therefore of particular interest. At this dose glucose absorption was unimpaired but sodium and water absorption were both reduced. Parallel abnormalities of sodium and water transport might have been anticipated as water uptake is dependent upon the osmotic gradient generated by active sodium transport.

Malabsorption after a single dose of MTX could be due either to a *direct* toxic effect on mature enterocytes or an *indirect* effect secondary to mitotic inhibition in crypt cells. Although the major action of MTX is on immature dividing cells it may also damage mature cells due to its effect on RNA and protein synthesis. This effect, however, is an early one and impaired enterocyte metabolism has been demonstrated within several hours of antifolate administration (Vitale, 1954). In the present study functional impairment was not seen 24 h after the highest dose and it therefore seems more likely that an indirect mechanism is involved.

A reduction in crypt mitotic activity has been previously demonstrated in jejunal biopsies after relatively low doses of MTX; $1$–$2\,mg\,kg^{-1}$. (Trier, 1962; Pinkerton *et al.*, 1982) but the cellular response to such inhibition is variable. This may range from a harmless delay in cell division to cell death and appears to depend upon both the metabolic state of the enterocyte and the stage of cell division at the time of drug exposure (Farber, 1971). Moreover cells damaged at crypt level may nonetheless migrate to the villus surface in the normal manner and although villus architecture is maintained a significant number of enterocytes may be functionally deranged. Alternatively, stasis of villus cell turnover could result with a failure to replace mature cells at the distal extrusion zone. The resultant increase in the number of "ageing" cells might reduce the absorptive efficiency without any gross structural change. Detailed stathmokinetic studies are required to determine precisely the effect of MTX on the migration pattern of crypt cells.

The persisting malabsorption 7 days after MTX is of particular interest as this may be comparable to the functional abnormalities found in patients several days after drug exposure. At this time gross structural abnormalities are no longer evident (Table I) and although subtle ultrastructural changes have been described in jejunal biopsies taken several days after MTX in such cases jejunal morphometry was similarly normal. (Gwevava *et al.*, 1981). A likely explanation for such persisting dysfunction is the repopulation of villi with relatively immature cells as a consequence of reactive crypt hyperplasia. Taminiau (1980) has demonstrated elevated thymidine kinase concentrations in rat jejunal mucosa during the recovery period and crypt hyperplasia has been described in man (Trier, 1962).

It is of note that glucose absorption was particularly affected both during the recovery period and after repeated low dose treatment. It is possible that repeated inhibition of crypt cell division over several weeks leads to a similar imbalance in villus cell turnover. The relative sparing of structure and function after biweekly low dose MTX by comparison with low daily or alternative day doses (Jolly & Fletcher, 1977; Baskerville & Batter-Hatten, 1977) could have been due either to the lower cumulative dose or to the greater time interval between doses. If insufficient time is allowed between doses the failure of mitotic recovery leads to crypt hypoplasia and villus atrophy. It is likely that the greater degree of xylose malabsorption in children receiving weekly rather than 3–4 weekly MTX is similarly due to the degree of mucosal recovery between doses (Pinkerton, 1981).

MTX metabolites and drug recycling may also contribute to prolonged enterotoxicity. Potentially toxic hepatic or intestinal metabolites may reach maximum concentration several days after a single dose or gradually accumulate with repeated doses. MTX polyglutamates have been shown to persist in various tissues for prolonged periods and could therefore influence enterocyte metabolism. (Jacobs *et al.*, 1975). Alternatively toxic concentrations of MTX may be present in the small gut as a result of enterohepatic recycling. Such a mechanism has been postulated as the cause of protracted diarrhoea in a child with leukaemia (Baird & Dossetor, 1981).

In conclusion it seems likely that MTX induced malabsorption is not, as is often assumed, simply a consequence of reduced villus surface area but involves the interaction of cell toxicity and alterations in villus population dynamics.

We are grateful to Mr B. Gregory and the staff of the animal house at the Institute of Child Health and to Ms F. Byron and Mr A. Phillips of the Dept. of Histopathology, Queen Elizabeth Hospital, Hackney.

**References**

BAIRD, G.M. & DOSSETOR, J.F.B. (1981). Methotrexate enteropathy. *Lancet*, **i**, 164.

BASKERVILLE, A. & BATTER-HATTEN, D. (1977). Intestinal lesions induced experimentally by methotrexate. *Br. J. Exp. Pathol.*, **58**, 663.

CRAFT, A.W., KAY, H.E.M., LAWSON, D.N. & McELWAIN, T.J. (1977). Methotrexate induced malabsorption in children with acute lymphoblastic leukaemia. *Br. Med. J.*, **ii**, 1511.

FARBER, E. (1971). Biochemical pathology. *Ann. Rev. Pharmacol.*, **11**, 71.

GWEVAVA, N.J.T., PINKERTON, C.R., GLASGOW, J.F.T., SLOAN, J.M. & BRIDGES, J.M. (1981). Small bowel enterocyte abnormalities caused by methotrexate therapy in acute lymphoblastic leukaemia of childhood. *J. Clin. Pathol.*, **34**, 790.

JACOBS, S.A., DERR, C.J. & JOHNS, D.G. (1975). Long term retention of 2,4 diamino methylpteroylglutamyl glutamate. *Proc. Am. Ass. Cancer Res.*, **16**, 65.

HARRIES, J.T. & SLADEN, G.E. (1972). The effects of different bile salts on the fluid, electrolytes and monosaccharides in the small intestine of the rat *in vivo*. *Gut*, **13**, 596.

JOLLY, L.E. & FLETCHER, H.P. (1977). The effect of repeated oral doses of methotrexate on its intestinal absorption in the rat. *Toxicol. Appl. Pharmacol.*, **39**, 23.

LEWIS, I.J., MAINWARING, D. & MARTIN, J. (1982). Enteropathy complicating maintenance therapy in acute lymphoblastic leukaemia. *Arch. Dis. Child.*, **57**, 663.

PINKERTON, C.R., GLASGOW, J.F.T., BRIDGES, J.M. & WELSHMAN, S.G. (1981). Enterotoxic effect of methotrexate; does it influence the drugs absorption in children with acute lymphoblastic leukaemia? *Br. Med. J.*, **282**, 1276.

PINKERTON, C.R., CAMERON, C.H.S., SLOAN, J.M., GLASGOW, J.F.T. & GWEVAVA, N.J.T. (1982). Jejunal crypt abnormalities associated with methotrexate therapy in children with acute lymphoblastic leukaemia. *J. Clin. Pathol.*, **35**, 1272.

ROBINSON, J.W.L., ANTONIOLI, J.A. & VANNOTTI, A. (1966). The effect of oral methotrexate on the rat intestine. *Biochem. Pharmacol.*, **15**, 1479.

SHAW, M.T., SPECTOR, M.H. & LADMAN, A.J. (1979). Effects of cancer, radiotherapy and cytotoxic drugs on intestinal structure and function. *Cancer Treat. Rev.*, **6**, 141.

TAMINIAU, J.A., GALL, D.G. & HAMILTON, J.R. (1980). Response of the rat small intestine epithelium to methotrexate. *Gut*, **21**, 486.

TRIER, J.S. (1962). Morphological alterations induced by methotrexate in the mucosa of human proximal intestine. *Gastroenterology*, **42**, 295.

VEHNO, V.M.K. (1976). Effect of methotrexate on drug absorption from the rat small intestine *in situ* and *in vitro*. *Acta. Pharmacol. Toxicol.*, **38**, 450.

VITALE, J.J., ZAMCHECK, N., DIGIORGIO, J. & HAGSTED, D.M. (1954). Effects of aminopterin administration on the respiration and morphology of the gastrointestinal mucosa of rats. *J. Lab. Clin. Med.*, **43**, 583.